# Complementary and Alternative Medicine Attitudes of Gynecologic Patients: Experience in a Tertiary Clinic

**DOI:** 10.1055/s-0041-1739462

**Published:** 2021-12-06

**Authors:** Ayçağ Yorgancı, Uğur Kemal Öztürk, Özlem Evliyaoğlu Bozkurt, Mesut Akyol, Ramazan Erda Pay, Yaprak Engin-Ustun

**Affiliations:** 1 Department of Obstetrics and Gynecology, Ministry of Health Ankara City Hospital, Ankara, Turkey; 2 Department of Obstetrics and Gynecology, İstanbul Zeynep Kamil Women and Child Diseases Education and Research Hospital, University of Health Sciences, Istanbul, Turkey; 3 Department of Obstetrics and Gynecology, Ankara Gülhane Education and Research Hospital, University of Health Sciences, Ankara, Turkey; 4 Department of Biostatistics and Medical Informatics, Ankara Yıldırım Beyazıt University, Ankara, Turkey; 5 Department of Obstetrics and Gynecology, Etlik Zübeyde Hanım Women's Health Education and Research Hospital, University of Health Sciences, Ankara, Turkey

**Keywords:** complementary medicine, alternative medicine, phytotherapy, gynecology, medicina complementar, medicina alternativa, fitoterapia, ginecologia

## Abstract

**Objective** To evaluate the knowledge, attitudes, and behaviors regarding complementary and alternative medicine methods of patients who were admitted to gynecology outpatient clinics.

**Methods** In the present survey, a questionnaire on complementary and alternative medicine practices was applied on 1,000 women (ages between 18 and 83 years old) who were admitted to the gynecology outpatient clinic of a tertiary maternity hospital. Demographic features and knowledge, attitudes, and behaviors about these methods were inquired in face-to-face interviews.

**Results** While 80.7% of the total participants thought that complementary and alternative medicine was beneficial, only 37.5% of them had used these methods previously. The rate of prior knowledge on this subject was of 59.7% and the source of information was physicians for 8.5% of the patients. However, 72.4% of all participants wanted to obtain information on these methods and 93.7% wanted to be informed by physicians. In the decision tree model, having knowledge about complementary and alternative medicine was the most effective factor determining its use ( *p* < 0.001). Phytotherapy was found to be the most used method, with 91.4%. The most preferred plant was onion (18.9%), and the most common reasons for herbal use were stress (15.4%) and fatigue (15.2%).

**Conclusion** More than one-third of the patients who applied to the gynecology outpatient clinics used one of the complementary and alternative medicine methods at least once. As gynecologists and obstetricians, we need to be more knowledgeable about these methods to provide correct guidance to our patients for accessing accurate and effective information.

## Resumo

**Objetivo** Avaliar o conhecimento, as atitudes e os comportamentos em relação aos métodos de medicina complementar e alternativa de pacientes internadas em ambulatórios de ginecologia.

**Métodos** Na presente pesquisa, um questionário sobre práticas de medicina complementar e alternativa foi aplicado a 1.000 mulheres (idades entre 18 e 83 anos) que foram admitidas nos ambulatórios de ginecologia de uma maternidade terciária. Características demográficas e conhecimento, atitudes e comportamentos sobre esses métodos foram investigados em entrevistas pessoais.

**Resultados** Enquanto 80,7% do total de participantes achavam que a medicina complementar e alternativa era benéfica, apenas 37,5% deles haviam usado esses métodos anteriormente. A taxa de conhecimento prévio sobre o assunto foi de 59,7% e a fonte de informação foi médica para 8,5% dos pacientes. No entanto, 72,4% de todos os participantes queriam obter informações sobre esses métodos e 93,7% queriam ser informados por médicos. No modelo de árvore de decisão, ter conhecimento sobre medicina complementar e alternativa foi o fator mais eficaz para determinar seu uso ( *p* < 0,001). A fitoterapia foi o método mais utilizado, com 91,4%. A planta preferida foi a cebola (18,9%), e os motivos mais comuns para o uso de ervas foram estresse (15,4%) e fadiga (15,2%).

**Conclusão** Mais de um terço das pacientes que se inscreveram no ambulatório de ginecologia utilizaram um dos métodos de medicina complementar e alternativa pelo menos uma vez. Como ginecologistas e obstetras, precisamos ter mais conhecimento sobre estes métodos a fim de fornecer orientações corretas aos nossos pacientes para o acesso a informações precisas e eficazes.

## Introduction

Complementary and alternative medicine (CAM) methods are a popular form of medicine all over the world. By definition, while complementary medicine is the treatment used by patients together with conventional treatments, alternative medicine is the treatment approach used instead of conventional treatments. 1 In an updated systematic review, the estimated rate of CAM use in the past 12 months ranged from 9.8 to 76%, with the highest prevalence in eastern Asian countries. 2 In Turkey, there are studies among cancer patients, and the frequency of CAM use among these patients varies between 22 and 84%. 3

Complementary and alternative medicine methods are generally divided into five categories: phytotherapy (herbals, special diets, etc.); alternative medicine (traditional Chinese medicine, acupuncture, Ayurveda, dry or wet cupping, etc.); mind-body therapies (meditation, hypnosis, biofeedback, etc.); external energy therapies (reiki, electromagnetic therapy, etc.); and body-based therapies (massage, chiropractic, etc.). 4

A small proportion of CAM methods have been scientifically tested, 5 while the remaining majority is widely used, despite not being proven. 6 Therefore, healthcare professionals should note the methods used by patients and obtain information about CAM applications. Scientifically proven methods that could be used alongside conventional treatments should be distinguished from other unsafe and scientifically unproven methods. It has been shown in most of the studies that women prefer these practices more often than men. 7 8 In addition, most CAM users do not notify their physicians about CAM use. 9 In this respect, the present survey was conducted to evaluate the knowledge, attitudes, and behaviors of women about CAM applications who were admitted to our gynecology outpatient clinics.

## Methods

The present survey was approved by the Ethics Committee of the Ankara Zekai Tahir Burak Women's Health Education and Research Hospital (77/2019). Informed consent was obtained from all the participants.

In the present cross-sectional research, a questionnaire on CAM practices was applied on 1,000 women who were admitted to the Ankara Zekai Tahir Burak Women's Health Education and Research Hospital Gynecology Outpatient Clinics between July and August 2019. The study was conducted in a tertiary maternity hospital and there were ∼ 10,000 admissions to gynecology outpatient clinics during the study period. Eligible patients were women aged ≥ 18 years old who could understand and speak Turkish without difficulty. Patients with an oncologic disease or any neurologic or psychiatric disease that might interfere with the understanding of questions and responses were excluded. The questionnaire included questions on age, education, residential area, income level, presence of chronic disease, whether they had information on CAM methods and the sources where they obtained this information, whether they found these applications useful, whether they used them before, if so what, how, and for what purpose they used them, whether they benefited from the CAM, and whether they would like to receive training about CAM and, if so, from whom. The questionnaire was filled out in person. Complementary and alternative medicine applications were divided into five subtitles: phytotherapy (herbals, special diets, etc.); alternative medicine (acupuncture, dry or wet cupping, Ayurveda, etc.); mind-body therapies (meditation, hypnosis, biofeedback, etc.); external energy therapies (reiki, electromagnetic therapy, etc.); and body-based therapies (massage, chiropractic, etc.). Each category and its examples were all written in common language that could be easily understood. There was another open-ended question under each category so that participants could enter unlisted CAM practices. For illiterate women, all the questions were read by one of the authors (Öztürk U. K.).

IBM SPSS Statistics for Windows version 22.0 (IBM Corp., Armonk, NY, USA) was used for statistical analysis. The suitability of age and CAM usage variables to normal distribution were examined with the Shapiro-Wilk test. The median (interquartile range [IQR]) value was given for the age variable that did not fit normal distribution. Age analysis between CAM users and nonusers was performed with the Mann-Whitney test. Descriptors of the questionnaires are shown in numbers and percentages. Crosstables were created for the necessary questions. The chi-squared test was applied to compare CAM use according to the place of residence, income, education, smoking behavior, and chronic disease. The number of total CAM methods used in chronic disease and smoking behavior was also analyzed with the Mann-Whitney test. The relationship between age and the number of total CAM methods used was evaluated with Spearman rank correlation (Rho). The chi-squared automatic interaction detection (CHAID) method was used to determine factors affecting the use of CAM. Data were analyzed at a 95% confidence level and *p* < 0.05 was considered to be significant.

## Results

The research was performed with 1,000 volunteers whose ages ranged from 18 to 83 years old, with a median age of 38.0 (IQR = 15.0). The demographic and clinical characteristics of the participants are given in Table 1 . While 37.5% ( *n* = 375) of the participants stated that they used one of the CAM methods at least once, 68.5% ( *n* = 257) of them used ≥ 2 CAM methods. The most preferred CAM method was phytotherapy (91.2%) followed by acupuncture (7.9%), detoxification (4.1%), wet cupping (2.4%), hypnosis (2.2%), ozone (2.2%), and leeches (0.5%). The median age of CAM users was higher than that of the nonusers (39.0 [IQR = 15.0] versus 36.0 [IQR = 16], Z = 3.191; *p* = 0.001, respectively). No significant relationship was found between the age of the participants and the number of total CAM methods used (Rho = - 0.072; *p* = 0.166). The percentage of CAM users was higher in the moderate-income group (41.0; χ ^2^ = 31.785; *p* < 0.001), and in women with chronic disease (44.5; χ ^2^ = 6.765; *p* = 0.009). There were no statistically significant differences in the percentage of CAM use according to residential area, education level, or smoking behavior (χ ^2^ = 1.975; *p* = 0.373; χ ^2^ = 4.188; *p* = 0.242; and χ ^2^ = 2.038; *p* = 0.153, respectively). The number of total CAM methods used was similar according to residential area, income status, educational level, smoking behavior, and the presence of chronic disease ( *p* > 0.05).

**Table 1:** Demographic and clinical characteristics of all participants and their distribution according to their opinion on the usefulness of complementary and alternative medicine modalities

	Total participants ( *n =* 1000)	CAM is useful ( *n* = 807)	CAM is not useful ( *n* = 193)	*p-value*
Age (median) (IQR)	38 (15)	38 (16)	35 (15)	0.044
Place of Residence (n,%) Provincial center District area Rural area	739 (73.9%) 228 (22.8%) 33 (3.3%)	590 (79.8%) 188 (82.5%) 29 (87.9%)	149 (20.2%) 40 (17.5%) 4 (12.1%)	< 0.001 < 0.001 < 0.001
Income (n,%) Low Moderate High	213 (21.3%) 688 (68.8%) 99 (9.9%)	188 (88.3%) 551 (80.1%) 68 (68.7%)	25 (11.7%) 137 (19.9%) 31 (31.3%)	< 0.001 < 0.001 < 0.001
Educational level (n,%) University High school Primary school Illiterate	218 (21.8%) 521 (52.1%) 216 (21.6%) 45 (4.5%)	164 (75.2%) 415 (79.7%) 188 (87.0%) 40 (88.9%)	54 (24.8%) 106 (20.3%) 28 (13.0%) 5 (11.1%)	< 0.001 < 0.001 < 0.001 < 0.001
Smoking behavior Yes No	343 (34.3%) 657 (65.7%)	272 (79.3%) 535 (81.4%)	71 (20.7%) 122 (18.6%)	< 0.001 < 0.001
Chronic disease (n,%)* No Yes Hypertension Diabetes mellitus Cardiac disease Pulmonary disease Other	755 (75.5) 245 (24.5) 86 (35.1) 56 (22.9) 24 (9.8) 40 (16.3) 73 (26.2)	591 (78.3%) 216 (88.2%) 76 (88.4%) 52 (92.9%) 21 (87.5%) 36 (90.0%) §	164 (21.7%) 29 (11.8%) 10 (11.6%) 4 (7.1%) 3 (12.5%) 4 (10.0%) §	< 0.001 < 0.001 < 0.001 < 0.001 < 0.001 < 0.001 NA
Information and source (n,%)* No Yes Physicians Internet Herbalist Newspaper + television Family and friends	403 (40.3%) 597 (59.7%) 92 (8.5%) 239 (22.2%) 126 (11.7%) 310 (28.8%) 310 (28.8%)	262 (65.0%) 545 (91.3%) 86 (93.5%) 216 (90.4%) 119 (94.4%) 273 (88.1%) 296 (95.5%)	141 (35.0%) 52 (8.7%) 6 (6.5%) 23 (9.6%) 7 (5.6%) 37 (11.9%) 14 (4.5%)	< 0.001 < 0.001 < 0.001 < 0.001 < 0.001 < 0.001 < 0.001
CAM use (n, %) No Yes	625 (62.5%) 375 (37.5%)	445 (71.2) 362 (96.5)	180 (28.8%) 13 (3.5%)	< 0.001 < 0.001
CAM methods (n, %)* Phytotherapy Acupuncture Detoxification Hypnosis Ozone Wet cupping Leeches	342 (91.2%) 33 (7.9%) 17 (4.1%) 9 (2.2%) 9 (2.2%) 10 (2.4%) 2 (0.5%)	333 (97.4%) 31 (93.9%) 16 (94.1%) 7 (77.8%) 8 (88.9%) 10 (100.0%) 2 (100.0%)	9 (2.6%) 2 (6.1%) 1 (5.9%) 2 (22.2%) 1 (11.1%) 0 (0.0%) –	< 0.001 < 0.001 < 0.001 NA NA NA NA

Abbreviation: CAM, complementary and alternative medicine.

* Since more than one answer can be given, the total may be different.

§: Rates have not been calculated since there are too many other chronic diseases.

**Fig. 1:**
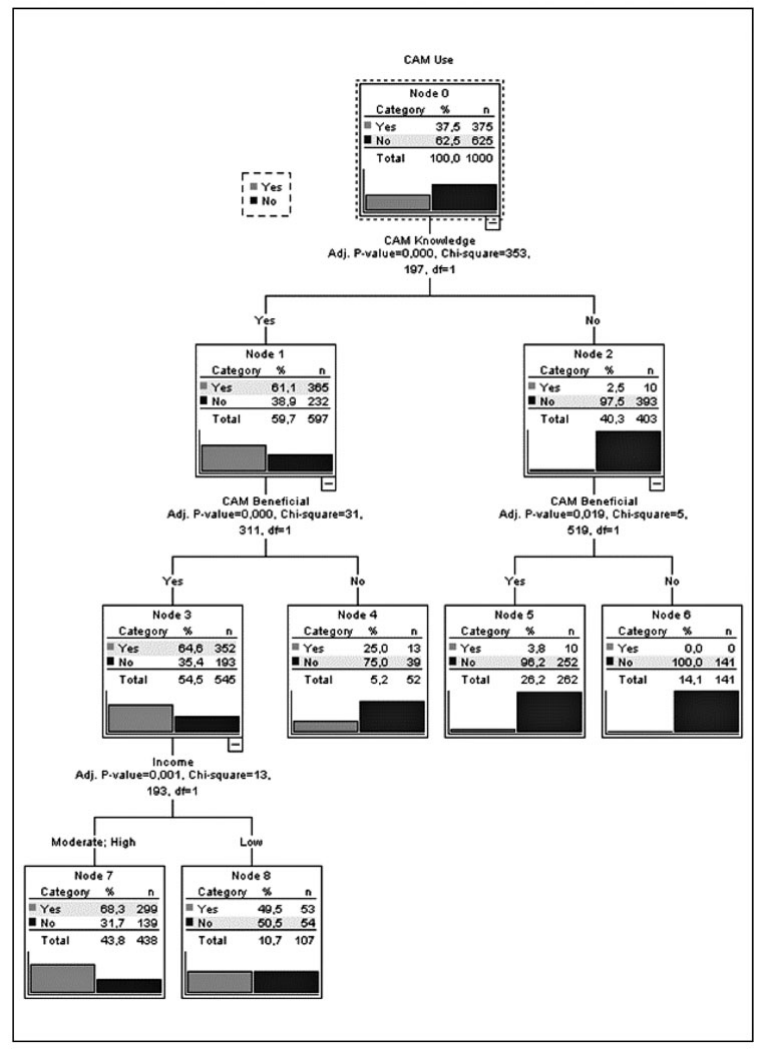
Decision tree of the Chi-squared Automatic Interaction Detection method used to determine the factors affecting the use of complementary and alternative medicine.

**Fig. 2:**
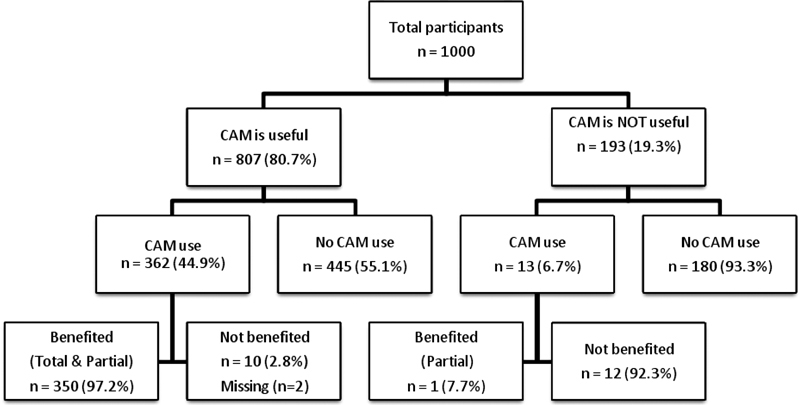
Flowchart of the study participants according to the opinions of the participants on the usefulness of complementary and alternative medicine, their percentages of use according to their opinions, and whether they benefited from complementary and alternative medicine or not.

When the factors affecting the use of CAM were examined with the CHAID method, having knowledge about CAM (χ ^2^ = 353.197; *p* < 0.001) was determined to be the most effective factor. Complementary and alternative medicine use was significantly higher among those who had information about CAM (χ ^2^ = 31.311; *p* < 0.001). Complementary and alternative medicine usage according to the factors is shown as a decision tree in Figure 1 . It was found that the established decision tree CHAID model correctly classified CAM users in 70.7% of cases and nonusers in 77.8%. The correct classification rate of the CHAID model was determined to be 78.5%. Figure 2 shows the opinions of the participants about the usefulness of CAM, the percentages of use according to their opinions, and whether they have benefited from CAM or not. While 80.7% of the participants thought that CAM was useful, only 44.5% of them stated that they have used CAM before. Table 1 also shows the demographic and clinical characteristics of women according to their opinion about the usefulness of CAM. Of the women who thought that CAM was not useful, 6.7% (13/193) had used CAM before. Of the women who used CAM, 40.8% stated that they have benefited from CAM, and 53.4% stated that they have partially benefited from it. Table 2 shows the benefit rates of different CAM modalities.

**Table 2:** Benefit rates of different complementary and alternative medicine modalities (n, %)

CAM Method	Benefited	Did not benefit	Total
Phytotherapy	333 (97.4%)	9 (2.6%)	342 (100.0%)
Acupuncture	31 (93.9)	2 (6.1)	33 (100.0%)
Detoxification	16 (94.1%)	1 (5.9%)	17 (100.0%)
Hypnosis	7 (77.8%)	2 (22.2%)	9 (100.0%)
Ozone	8 (88.9%)	1 (11.1%)	9 (100.0%)
Wet cupping	10 (100.0%)	0 (0.0%)	10 (100.0%)
Leeches	2 (100.0%)	0 (0.0%)	2 (100.0%)

Abbreviation: CAM, complementary and alternative medicine.

Phytotherapy was the most preferred CAM method. The most common herbals were onion (18.9%) followed by black seed (12.3%), ginger (11.6%), garlic (11.4%), and stinging nettle (6.1%). St. John's wort, yarrow, echinacea, and the fruit of *vitex agnus-castus* were used by < 5% of the respondents; however, there were a total of 60 different herbs (mixed herbal tea, sage, parsley, aloe vera, flaxseed etc.) among the answers given ( Table 2 ). Out of the 336 patients that used phytotherapy, 133 (39.5%) of them used ≥ 2 herbals in various forms.

When the plants used in phytotherapy were examined according to their intended use, the herbals with a use frequency of < 3% were included under the title of others ( Table 3 ). The most common purposes of use were stress (15.4%) and fatigue (15.3%) followed by weight problems (14.5%), menstrual irregularities (13.0%), and body care (11.1%). Phytotherapy was used due to ovarian cysts for 6.8%, while the percentage of use due to uterine leiomyoma and sexual health were 3.3% for each. Phytotherapy was used for a total of 40 different reasons.

**Table 3:** Plants used in phytotherapy, use rates, and forms

Plant	*n*	%	The most common form of use
Onion	112	18.9	Boiling then drinking the water
Black seed	73	12.3	In oil form; with yogurt, honey, molasses
Ginger	69	11.6	As a tea; in food and salad
Garlic	68	11.4	Boiling then drinking the water; with lemon; over the skin and hair
Stinging nettle	36	6.1	Boiling then drinking the water; in food and salad
St. John's wort	23	3.9	In oil form; as a tea
Yarrow	19	3.2	As a tea; with fig
Echinacea	15	2.5	As a tea
Fruit of *vitex agnus-castus*	14	2.4	Boiling then drinking the water
Others	165	27.8	Boiling then drinking the water; as a tea; in food and salad; in oil-cream form
Sum*	594	100.0	

* Since more than one answer can be given, the total may be different.

A total of 72.4% of the participants stated that they would like to get information on CAM methods, while 27.6% stated that they did not want to. Of the women who wished to get information, 65.6% of them wanted to get information on phytotherapy, followed by acupuncture (20.4%), hypnosis (14.5%), detoxification therapy (12.9%), and ozone therapy (10.7%). They wished to be informed by physicians (93.8%), followed by newspapers and television (17.7%), internet (9.8%), and herbalists (6.1%) ( Table 4 ).

**Table 4:** Purposes and rates of use of herbs used in phytotherapy *n* (%)

Reason	Onion	Black seed	Ginger	Garlic	Stinging nettle	Others	Total
Stress	39 (17.8)	36 (16.4)	31 (14.2)	30 (13.7)	19 (8.7)	64 (29.2)	219 (15.4)
Fatigue	34 (15.7)	32 (14.7)	35 (16.1)	35 (16.1)	18 (8.3)	63 (29.0)	217 (15.3)
Weight problems	40 (19.3)	33 (15.9)	29 (14.0)	31 (15.0)	12 (5.8)	62 (30.0)	207 (14.5)
Menstrual irregularities	56 (30.3)	24 (13.0)	16 (8.6)	18 (9.7)	5 (2.7)	66 (35.7)	185 (13.0)
Body care	23 (14.6)	22 (14.0)	17 (10.8)	26 (16.6)	12 (7.6)	57 (36.3)	157 (11.1)
Ovarian cyst	44 (45.8)	10 (10.4)	8 (8.3)	11 (11.5)	4 (4.2)	19 (19.8)	96 (6.8)
Uterine leiomyoma	15 (31.9)	5 (10.6)	6 (12.8)	5 (10.6)	5 (10.6)	11 (23.4)	47 (3.3)
Sexual health	4 (8.5)	6 (12.8)	4 (8.5)	3 (6.4)	2 (4.3)	28 (59.6)	47 (3.3)
All other uses	46 (18.7)	50 (20.3)	55 (22.4)	58 (23.6)	28 (11.4)	158 (64.2)	246 (17.3)
Total*	301 (21.2)	218 (15.3)	201 (14.1)	217 (15.3)	105 (7.4)	528 (37.2)	1421 (100.00)

* Since more than one answer can be given, the total may be different.

## Discussion

Although CAM is widely used all over the world, the use rates and the methods of CAM differ among countries due to social, cultural, and economic factors. Sociodemographic features such as age, gender, education level, income, and health status have all been found to be different in studies conducted around the world. In Europe, the rate of CAM use was reported as ranging from 10 to 40% across countries and was more common among women, people with higher education, and those with good health. In addition, the nature of health problems and income status were found to affect CAM methods. 10 According to the results of a national survey in the United States, single CAM use was common among adults, and CAM practices differed by age, gender, ethnic descent, and educational background. 8 In Japan, the prevalence of CAM use was 62.1% and female gender and health anxiety were found to be associated with higher CAM use. 11 In our study, we aimed to evaluate knowledge, attitudes, and behaviors about CAM practices of women admitted to our gynecology outpatient clinic. The results of our study showed that 37.5% of the women used CAM before and that CAM use was more common in women with moderate income and in those who had a chronic disease. The median age of CAM users was 39 years old, which was higher than that of nonusers. Similarly, Sarris et al. 12 reported that middle-aged women (aged between 40 and 64 years old) used any kind of CAM significantly more than older women (≥ 65 years old).

Considering the frequent use of CAM in the reproductive age of women, and considering that this use is generally without the knowledge of healthcare professionals, 13 both obstetricians and gynecologists should be careful and vigilant about CAM use. 14 In Germany, the rate of CAM use was of 61.4% among third-trimester pregnant women. 15 The use and interactions of CAM are extensively researched especially among gynecologic oncology patients. 16 17 18 In adolescents, primary dysmenorrhea draws attention as the primary reason. 19 In a survey, women were asked to report the use of ≥ 1 CAM methods especially for the treatment of obstetric or gynecologic problems, and the reported rate was of 54.5%. 20 In our study, we asked the reason for CAM use without any restriction, and to the best of our knowledge, there is no study in the literature reflecting all reasons for the gynecological patient group. In Turkey, most of the studies have been conducted among gynecological cancer patients. The frequency of CAM use in these studies varies between 47 and 61%. 21 22 23 However, in the study of Turkish women with urinary incontinence, the frequency of CAM use was found to be very low, at 7%. 24

Our results showed that the most common sources of information on CAM methods were newspapers/television and family/friends. Similar results have been reported from different countries. 16 20 25 In our study, the least common source of information about CAM was physicians, with 8.5%, although having knowledge about CAM was determined as the most effective factor in CAM use. In the aforementioned studies, the rate of physicians as a source of information ranged from 6.4 to 15.7%. 16 20 25 In another study from our country, Kav et al. 3 found that 10% of the patients received information from a physician. The main conclusion arising from these studies is that health professionals, especially physicians, tend not to inform patients on this subject. Also, > 90% of the participants in our survey stated that they wanted to get information from physicians. In this regard, Münstedt et al. 26 emphasized that information about CAM applications is rarely given in universities, especially in medical faculties, and that healthcare professionals must obtain this information from other places.

In our study, the most used CAM method was phytotherapy, with 90%. As stated, due to sociocultural and socioeconomic factors, the preferred method of CAM varies considerably between countries. Yet, in the study by Sirois et al., 27 phytotherapy stands out as the most frequently used method. In other studies conducted with gynecologic cancer patients in our country, phytotherapy was found to be the most used CAM method. 21 22 23 We determined that 60 different herbals were used by the participants and that the most popular was onion, followed by black seed. Onion has been shown to have antioxidant, anticarcinogenic, hypolipidemic, hypoglycemic, and antiaggregatory effects. 28 The reason for onion being the most preferred herb might be the popularity of discourses on onion cures, especially in visual media in Turkey nowadays. Nevertheless, these preferences differ from oncological patients, since stinging nettle was the preferred herbal for cancer patients in our country. 21 22 29 On the other hand, even pregnant women do not worry about using herbal remedies, as they feel safe in using them. 30 Pallivalapila et al. 15 reported that 40 different plant varieties have been used by 62% of pregnant women in the 3 ^rd^ trimester. Thus, the point that must be taken into consideration is the possible negative interactions between prescribed drugs and herbs. For example, black seed, the second most preferred herbal in our study, is an herb known to have antidiabetic properties. 31 However, it was claimed to cause acute renal failure in a diabetic woman. 32 Another interesting finding of our study was that approximately one-fourth of women used phytotherapy because of gynecologic problems, including menstrual irregularities (13%), ovarian cysts (6.8%), uterine leiomyoma (3.3%), and sexual health (3.3%). In Germany, the gynecological reasons for gynecologists to recommend phytotherapy were postmenopausal symptoms (63%), premenstrual symptoms (56%), infertility (23%), genital inflammation (22%), incontinence (18%), polycystic ovary syndrome (16%), and uterine leiomyoma (12%). 26

The major limitation of the present survey was that it was conducted in a single center. The results of the present survey cannot be attributed to the general population of women in Turkey. However, the survey was conducted in one of the largest maternity hospitals of our country. Additionally, it has the advantage of having a larger number of participants compared with similar studies in the literature and having participants fill out the questionnaire in person.

## Conclusion

The results of our survey have shown that more than one out of every three women who applied to the gynecology outpatient clinic have used a CAM method at least once before. The most used CAM method was phytotherapy, which can be attributed to the stereotypical and conservative view that might be based on traditional experience lasting for centuries. Considering that the most effective factor in the use of CAM is to have knowledge about these methods and the most desired source of information on these methods is physicians, we gynecologists and obstetricians have more responsibilities. We need to be more knowledgeable of these methods to provide correct guidance for women to access accurate and effective information.
